# Vitamin D Deficiency in Both Oral and Systemic Manifestations in SARS-CoV-2 Infection: Updated Review

**DOI:** 10.3390/medicina59010068

**Published:** 2022-12-28

**Authors:** Alin Constantin Pinzariu, Ivona Andreea Sova, Minela Aida Maranduca, Nina Filip, Ilie Cristian Drochioi, Calin George Vamesu, Andreea Clim, Loredana Liliana Hurjui, Mihaela Moscalu, Radu Petru Soroceanu, Dragomir Nicolae Serban, Ionela Lacramioara Serban

**Affiliations:** 1 Department of Morpho-Functional Sciences II, Discipline of Physiology, “Grigore T. Popa” University of Medicine and Pharmacy, 700115 Iasi, Romania; 2 IOSUD Faculty of Medicine, “Grigore T. Popa” University of Medicine and Pharmacy, 700115 Iasi, Romania; 3 Internal Medicine Clinic, “St. Spiridon” County Clinical Emergency Hospital, 700115 Iasi, Romania; 4 Department of Morpho-Functional Sciences II, Discipline of Biochemistry, “Grigore T. Popa” University of Medicine and Pharmacy, 700115 Iasi, Romania; 5 Department of Oral and Maxillofacial Surgery and Reconstructive, Faculty of Dental Medicine, “Grigore T. Popa” University of Medicine and Pharmacy, 700020 Iasi, Romania; 6 Department of Preventive Medicine and Interdisciplinarity, “Grigore T. Popa” University of Medicine and Pharmacy, 700115 Iasi, Romania; 7 Department of Surgery I, Discipline of Surgical Semiology, “Grigore T. Popa” University of Medicine and Pharmacy, 700115 Iasi, Romania

**Keywords:** coronavirus, COVID-19, SARS-CoV-2, vitamin D, prevention, systemic manifestations

## Abstract

The specialized literature emphasizes the fact that vitamin D has a potentially beneficial effect in the context of the current COVID-19 pandemic. The purpose of this article is to highlight the role of vitamin D, both prophylactic and curative, in the treatment of patients diagnosed with COVID-19. Even though its relevance is still unknown and causes various controversies, there is currently no specific treatment for patients diagnosed with COVID-19. There are various prevention strategies with new vaccination schedules, but additional randomized and clinical trials are still needed to combat this pandemic. In addition to the systemic manifestations of SARS-CoV-2 infection, oral manifestations of this disease have also been described in the literature. The etiology of oral manifestations associated with COVID-19 infection and vitamin D deficiency remains controversial. In the present studies, oral manifestations such as salivary gland infections, aphthae, erythema, gingivitis, ulcers, etc. have been reported. This is a new topic, and the prevalence of manifestations is described in only a few studies, which is inconsistent with the number of COVID-19 cases reported since the beginning of the pandemic. The clinical symptomatology in patients with current COVID-19 infection is polymorphic. Whether the oral manifestation is directly caused by SARS-CoV-2 or a secondary manifestation remains an important topic to analyze and discuss.

## 1. Introduction

SARS-CoV-2 infection has become a global public health problem causing millions of deaths. The first epidemic to include a respiratory syndrome was (SARS)-CoV, first described in 2002 in China, and later Middle East (MERS)-CoV was reported in 2012 in Saudi Arabia. In late 2019, the first cases of CoV were confirmed in Wuhan, Hubei, China; originally named 2019-nCoV, it was later renamed by the World Health Organization to COVID-19, in February 2020. The main cause of death from infection with COVID-19 is generally due to severe atypical pneumonia and associated comorbidities [1,2].

Currently, although there is no specific treatment to stop the pandemic except for the vaccine and the prevention measures that were enforced, studies emphasize the particularly important roles of micronutrients on the immune system.

Current data in the literature, although there are conflicting studies, have highlighted the benefits of micronutrient supplementation that can modulate the immune system (vitamin C, D, and zinc according to studies) and decrease the risk of infection, hospitalization, and death rate among patients diagnosed with COVID-19 [3].

## 2. Materials and Methods

The literature search was conducted in PubMed database (accessed on 1 September 2022), focusing on studies published within the last 11 years. Our search queried “vitamin D [AND] oral manifestation [OR] systemic manifestations [OR] COVID-19 [AND] SARS-CoV-2” and was limited only to prospective and retrospective studies and metanalyses, omitting abstracts, documents, and reviews. Our search resulted in 87 total references. These were manually reviewed, and only 25 references were within our scope of interest ranging from 2013 to 2022.

## 3. Structure, Sources, and Vitamin D Absorption

The amount of sunlight needed to meet our vitamin D requirements is difficult to estimate because it depends on many important factors, such as skin pigment, age, season or time of day, and not least altitude [4,5]. Because foods are deficient in natural sources of vitamin D, diet does not provide the necessary dose of vitamin D for most people. Therefore, in many situations where there is inadequate exposure to the sun as well as low food intake, vitamin D supplementation may be necessary. A suitable example of this situation is represented by the restrictions imposed by the pandemic caused by COVID-19. This restriction affects the majority of the population by reducing their exposure to sunlight and, consequently, the synthesis of vitamin D.

There are two main dietary sources of vitamin D: cholecalciferol (vitamin D3) and ergosterol (vitamin D2). From a chemical point of view, vitamin D is a cholesterol derivative, vitamin D2 is obtained by ergosterol oxidation of plant origin, while vitamin D3 is obtained by 7-dehydrocholesterol oxidation of animal origin. Foods of animal origin in which we find vitamin D3 are tuna liver oil, salmon, beef liver, and mackerel. Mushrooms are the food of plant origin in which we find vitamin D2 (for example the mushroom *Agaricus bisporus*) [5,6,7].

Vitamin D esters (either vitamin D3 or D2) are hydrolyzed by pancreatic esterases to release vitamin D, which are emulsified in the presence of bile salts and pancreatic juice, thus forming mycelia that diffuse into enterocytes [4]. With the diffusion of mycelia in the jejunum, the formation of chylomicrons occurs, which initially pass into the lymph and then into the blood. In the blood, vitamin D is bound to α-globulin (VDBP-vitamin D-binding globulin) and thus transported to the liver. As only a fraction is contained in the chylomicron, vitamin D can be absorbed by adipose tissue and skeletal muscle tissue [5,8]. The rest of the chylomicrons that transport vitamin D to the liver, with the help of the vitamin D-binding protein (VDBP), will make it possible to enter the hepatocytes and, subsequently, facilitate their transport to the various tissues that need them [6,8].

Cholecalciferol (Vitamin D3) is formed at the integumentary level, being photosynthesized in the skin as a result of the irradiation of 7-dehydrocholesterol (a substance found physiologically in skin tissue) by ultraviolet solar rays [6]. Therefore, adequate exposure to the sun prevents vitamin D deficiency. Vitamin D, which enters the body exogenously (through food intake), is identical to cholecalciferol formed in the skin, except for substitutions at one or more atoms, which do not affect its function [7].

Cytochrome P450 mixed-function oxidases (CYPs) have a major role in vitamin D metabolism, 25-hydroxylation, and 1α-hydroxylation, with localization either in the endoplasmic reticulum (ER) (e.g., CYP2R1) or mitochondria (e.g., CYP27A1, CYP27B1, and CYP24A1). The electron donor for ER enzymes is reduced NADPH-dependent P450 [6,9,10].

## 4. Liver and Renal Metabolism of Vitamin D

**Figure 1:**
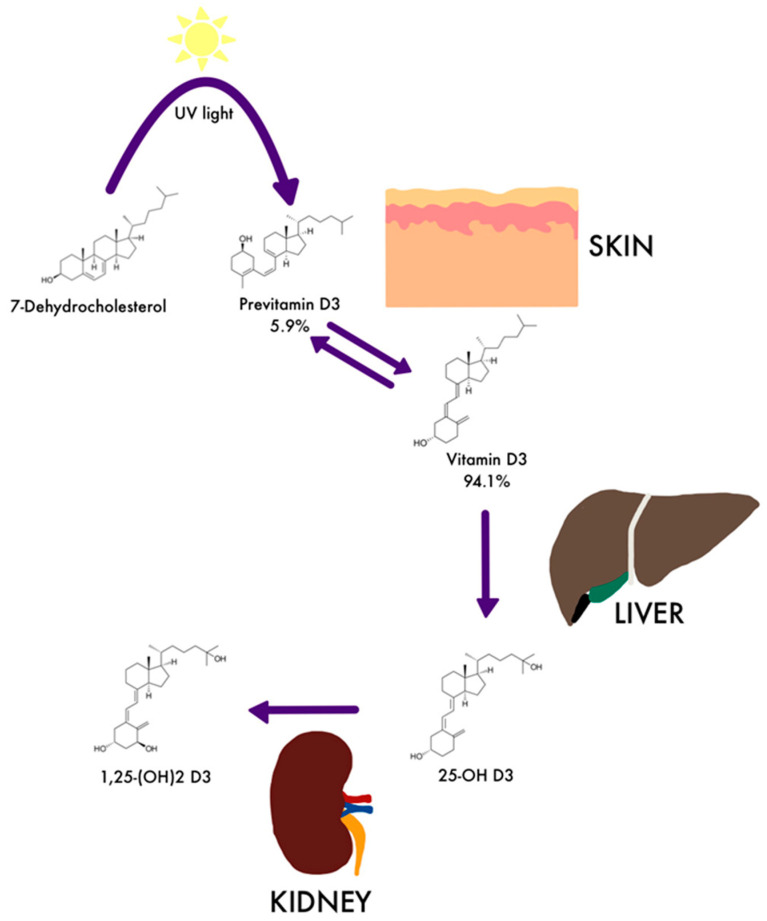
Vitamin D, liver, and renal metabolism (Adapted with permission from [12]).

The first step in the activation of cholecalciferol is its transformation into 25-hydroxycholecalciferol under the action of 25-hydroxylase (CYP450 enzyme, especially CYP2R1), which occurs in the liver within 24 to 96 h [6,10]. The process is limited because 25-hydroxycholecalciferol exerts a feedback inhibitory effect on the conversion reaction. Monohydroxylated derivatives can follow three paths in the body (Figure 1): they carry out the hepato-entero-hepatic circuit; they are hydroxylated in the liver under the action of 24-hydroxylase with the formation of 24,25-dihydroxy-vitamin D2 or D3, biologically inactive forms, but which can stimulate 25-hydroxy-1α-vitamin D hydroxylase from the renal level; and the transport to the kidneys is achieved by binding to the specific globulin VDBG, where in the mitochondria of the cells of the proximal renal tubules they are hydrolyzed to obtain the active form 1α, 25 dihydroxyvitamin D (calcitriol), dependent on CYP450 (CYP27B1) and tightly regulated by calcium levels and blood phosphate via PTH and fibroblast growth factor 23 (FGF-23) [11].

The conversion of 25-hydroxycholecalciferol to 1,25-dihydroxycholecalciferol takes place in the presence of the parathyroid hormone. In its absence, the amount of 1,25-dihydroxycholecalciferol is almost zero [6,13,14]. Therefore, the functional effects of vitamin D on the body are due to PTH. Since the final synthesis of vitamin D occurs in the body, from dietary precursors, calcitriol can be considered a hormone, since it does not meet all the conditions to be considered a vitamin [12,15,16].

## 5. Effects of Vitamin D

The mechanism of action of calcitriol is mediated by VDR, known as vitamin D receptor, which belongs to a subfamily of nuclear receptors that act as transcription factors in target cells after forming a heterodimer with the retinoid X receptor (receptor for retinoic acids) with an affinity approximately 1000 times higher than for 25-hydroxycholecalciferol, which explains the different intensity of their biological effects [17,18,19].

Vitamin D has a well-known role in the homeostasis of the osteoarticular system and is essential for good calcium absorption. Being essential for a good absorption of calcium, there has been increased interest in the involvement of vitamin D in the metabolic pathways involved in muscle function.

Vitamin D is a key nutrient in maintaining the health of the musculoskeletal system, and its deficiency can lead to myopathy, a disorder characterized by hypotonia, atrophy of skeletal muscles, and asthenia [20,21,22].

Muscle biopsies obtained from vitamin D-deficient adults show the presence of large interfibrillar spaces, fibrosis, and a loss of type II fibers. There is also an increase in fat infiltration into muscle, a similar effect seen in the elderly, where there is a progressive loss of muscle mass, muscle contraction force, and an increase in transdifferentiated adipose tissue [23,24].

## 6. Risk of Vitamin D Deficiency

Vitamin D deficiency in all age groups is a widespread phenomenon globally, becoming a public health problem mainly caused by low sun exposure. It is estimated that more than one billion people suffer from vitamin D deficiency, a number that increases with age and associated comorbidities [25,26,27]. Studies showing the risk of vitamin D deficiency are summarized in Table 1.

It has been shown that vitamin D administration is associated with the recovery and increased quality of life in patients after bone surgery. Following postoperative supplementation with vitamin D, it was observed that pain in the skeletal system decreased in intensity [22,23,33,34].

Rabbits and mice with experimentally induced vitamin D deficiency show low insulin secretion, and following vitamin D supplementation, a normalization of insulin secretion is observed [28,35,36,37]. Mice with abnormalities in the specific receptor for vitamin D have been found to have reduced glucose tolerance compared to animals that have this functional receptor.

Data from the literature highlight the fact that fat infiltration in muscle tissue not only has a direct impact on muscle functionality and strength, but is also an important independent risk factor for metabolic diseases such as insulin resistance and diabetes [23,38,39,40].

Several studies have demonstrated that myogenic precursor cells retain the potential for transdifferentiation towards the adipogenic lineage. Previous studies have revealed that vitamin D has potent effects on adipogenesis and myogenesis [29,40,41].

Experimental research has shown that a hyperlipidemic, vitamin D-deprived diet is associated with reduced femur mineral density compared to animals fed a high-fat diet, suggesting that vitamin D influences bone metabolism [42,43,44,45].

Experimental studies on laboratory animals have demonstrated an interdependence between vitamin D supplementation and the degree of bone repair. It should be noted that obesity, smoking, severe anemia, and diabetes will prevent bone regeneration associated with vitamin D deficiency, suggesting the particularly important role of vitamin D in the body [20,23,46,47,48]. The molecular mechanisms responsible for the differentiation and evolution of human adipogenesis are not fully elucidated, especially because of the difficulty in identifying and characterizing human adipocyte precursors.

Preadipocytes—those corresponding to unilocular cells, but also to multilocular cells—are difficult to identify, due to the impossibility of their differentiation, in relation to any other fibroblast-like cell [49,50].

Recent evidence suggests that vitamin D, in addition to its classic role in calcium homeostasis and maintenance of the bone system, has also anti-inflammatory and antithrombotic effects.

Vitamin D deficiency influences the secretion of proinflammatory cytokines and chemokines, and there is clear evidence of the severity of viral respiratory infections that, in the long term, increases the risk of developing hypertension, diabetes, congestive heart failure, and peripheral arterial disease, such as myocardial infarction, stroke, and death [51,52,53].

Slightly low plasma levels of vitamin D (approximately 25 ng/mL) increase the risk of patients developing hypertension, and at values of 3–4.8 ng/mL, much reduced, they are associated with an increased risk of heart disease, accident ischemic stroke, myocardial infarction, and early death by 40%, 64%, and 57%, respectively, compared with individuals with plasma vitamin D levels of 18.83–28.44 ng/mL [30,31,54,55,56]. The functioning and integrity of skeletal muscles is influenced by the development of osteoporosis, as a result of vitamin D deficiency [45,57].

Although there is insufficient scientific evidence, vitamin D may play an important role in tumor progression in the oral field [58]. In a case-control study, VDD was associated with an increased risk of esophageal squamous cell carcinoma at the oropharyngeal level and with an increased prevalence in tobacco and alcohol-consuming patients [32,59]. Another study concluded that in premalignant and malignant conditions there is an increase in VDR expression and the administration of vitamin D had the effect of significantly reducing toxicity, morbidity, and increasing the quality of life-related to treatment in advanced oral cancer [17,60,61].

Patients diagnosed with SARS-CoV-2 present specific or non-specific clinical symptoms, symptomatic or asymptomatic, and they can affect the systemic organism. Recent studies have also described the oral manifestation of COVID-19 infection in these patients. The SARS-CoV-2 virus was detected in the patient’s saliva and which could cause lesions at this level, through inflammatory effects and tissue destruction produced at the oral lesions [62]. Lesions such as gingivitis, erythema, aphthous, salivary gland infections, and ulcers were described in new studies [63]. The etiology involved in oral manifestation associated with the infection with COVID-19 remains an interesting subject to discuss. There are not enough studies to confirm that these lesions are directly caused by SARS-CoV-2 or that this could be associated with SARS-CoV-2 infection correlated with the favorable factors. The current studies have considered that the presence of poor oral hygiene, smoking, existing dental conditions such as periodontal disease, comorbidities, and adverse reactions to medication administered were associated with oral lesions [64].

Additionally, the data from the literature reported that the presence of secretory dysfunction of the salivary glands and acute parotitis can be initial clinical symptoms of COVID-19.

The prevalence of oral lesions of these patients is analyzed in few studies, which is discrepant with the number of cases reported with COVID-19 since the beginning of the pandemic.

## 7. Vitamin D and COVID-19 Infection

The pandemic caused by the SARS-CoV-2 infection has registered numerous deaths among the population and controversies worldwide.

The incidence of respiratory tract infections (RTIs) is more common in winter, especially in northern regions, compared to the summer months. This is also true for the rapid global spread of the infectious disease in 2019 of coronavirus disease (COVID-19) during the winter period, which later became a pandemic, because the virus is more easily transmitted in cold temperatures [12]. Based on the premise that insufficient intake of vitamin D3 may contribute to the development and severity of the infection with COVID-19, in order to combat this pandemic, it is considered that the administration of an adequate amount of vitamin D3 may be effective in keeping the pandemic under control until it develops an effective therapy and chemoprophylaxis [65,66].

To date, there are very few studies on the link between vitamin D and COVID-19. Studies showing the relationship between vitamin D and COVID-19 are summarized in Table 2.

Daily supplementation with Vit D (1000 to 3000 IU) to maintain serum levels within normal limits may be beneficial both in the prevention and treatment of infection caused by COVID-19 [84].

The studies carried out before the COVID-19 pandemic—a study carried out on 5660 participants (age ranging from 6 months to 75 years) as well as a study carried out on 10,933 participants (aged 0–95 years) from 14 different countries—demonstrated that supplementation with vitamin D offers a protective and effective role in reducing the risk respiratory tract infections [12,85].

Daily supplementation with moderate doses of vitamin D prevents the occurrence of multisystem injuries induced by COVID-19 infection, mortality, coagulopathy, and even reduces the risk and severity of COVID-19 [80,81,82,83,86], offering benefits among these patients.

Retrospective studies to date have demonstrated a relationship between serum vitamin D3 levels and respiratory tract infections.

For example, the authors of a preliminary study conducted among the patients with COVID-19 found that severity of infection was correlated with serum vitamin D levels. The authors found that 85.5% of patients with serum levels of Vit D3 above 30 ng/mL presented a moderate form of the disease, while 72.8% of patients with Vit D3 deficiency (<20 ng/mL) had a severe form [87,88].

The authors of a study of approximately 191,779 patients from 50 states, with an average age of approximately 54 years, in which vitamin D values were correlated in patients diagnosed with SARS-CoV-2, found that the positivity rate for hypovitaminosis D was higher among 39,190 patients compared with 27,870 patients with normal vitamin D values [89].

According to a study in a group of 178 patients from Indonesia, where it was investigating the correlation between the serum level of Vit D3 and the severity of the COVID-19 infection, among patients with serum levels of Vit D3 between 20–30 and <20 ng/mL, deaths were approximately 10.12 times higher compared to the group of patients with serum levels within normal limits [90].

At the same time, another limited cohort study (among 43 cases) from Singapore highlighted the fact that in the case of patients with COVID-19, the administration of the oral dose of vitamin D3 (1000 IU), Mg (150 mg), and vitamin B12 (500 μg) significantly reduced the administration of oxygen therapy, compared to the control group [91].

Thus, vitamin D has proven to be an essential prevention factor against respiratory infections. Vitamin D deficiency was observed in patients with chronic pathologies and generally had a poor prognosis. In elderly patients, severe hypovitaminosis D was found to be an independent predictor of community-acquired pneumonia, and death. Additionally, it was associated with lung inflammation, causing acute respiratory distress syndrome (ARDS), damage to the respiratory epithelium, and hypoxia. Moreover, considering the increased incidence of pulmonary fibrosis—sequelae of the infection caused by COVID-19—it is important to remember that vitamin D prevents a profibrotic phenotype caused by TGF-1 of the lung cells [92,93]. Several studies emphasize the need for adequate management of oral conditions in patients with diabetes to avoid inflammatory conditions and potential morbidities [94]. Significant associations were identified between vitamin deficiency D and glucose, with this aspect negatively influencing the evolution of patients with COVID-19 [95].

Current available literature data provide information on the importance of micronutrient administration in the management, evolution, and prognosis of COVID-19.

The pathophysiological mechanisms by which vitamin D deficiency may cause progression from simple lesions (airway inflammation and diffuse alveolar lesions) to complicated, clinically significant lesions (vascular inflammation and thrombosis associated with COVID-19), and the lack of clinical and cohort studies on the role of vitamin D3 in the prevention of infection with COVID-19 present areas of future research, both with a therapeutic and preventive role [78,89]. Endothelial dysfunction is the central element of the pathology triggered by the SARS CoV-2 virus.

## 8. The Role of Vitamin D in Endothelial Dysfunction

In the literature, the important role of endothelial dysfunction in patients with COVID-19 and several interrelated mechanisms involving the renin-angiotensin system and immune cells have been proposed and also analyzed and discussed [96,97,98].

Endothelial dysfunction has a particular role in vascular inflammation associated with COVID-19 infection and coagulopathy [91,99,100,101], but the role of endothelial dysfunction in patients with vitamin D deficiency and COVID -19 remains controversial [95].

The vascular endothelium is essential for maintaining regulating vascular tone and vascular homeostasis. It is responsible for the regulation of oxidative stress by releasing mediators such as nitric oxygen (NO), prostacyclin, endothelin, and controlling the local activity of angiotensin II [99].

Endothelial dysfunction is characterized by alteration of the regulatory functions of the endothelium, causing an imbalance between relaxing and contracting factors, procoagulant and anticoagulant mediators, or substances that inhibit and promote growth [102].

Pathophysiological mechanisms underlying endothelial dysfunction include risk factors for cardiovascular disease such as hypertension, insulin resistance, dyslipidemia, smoking, hyperhomocysteinemia, or a combination of these factors [53].

Reduction of nitric oxide (NO) production and/or bioavailability is considered the central mechanism responsible for endothelial dysfunction [91,94,103]. The stability of NO is dependent on several factors that prevail in the physiological environment, such as oxygen-derived free radicals (superoxide), pH, and thiol group availability. Thiols stabilize NO by reducing available oxygen-derived free radicals. Another particularly important role in the occurrence of endothelial dysfunction is the serum level of homocysteine, which is considered an independent risk factor in the occurrence of cardiovascular pathology, including hypertension.

The role of the endothelium as a target of vitamin D is demonstrated by the direct effects of vitamin D on endothelial function. It prevents endothelial cell death by modulating apoptosis and autophagy through multiple mechanisms including inhibition of superoxide anion generation and induction of NO production. The release of NO induced by vitamin D during oxidative stress (imbalance between pro-oxidants and anti-oxidants) provides a protective role for cells.

The multiple properties of NO (vasodilator, antiplatelet, anti-proliferative, anti-adhesive, decreased permeability, and anti-inflammatory), NF-κB activation by pro-inflammatory genes and oxidative stress play an important role in endothelial dysfunction and cell apoptosis [101,103,104]. Endothelial apoptosis is associated with NO and peroxynitrite, through the action of NO on vascular homeostasis and endothelium-derived contractile factors such as angiotensin II and endothelin-1.

Previous studies have suggested that vitamin D protects against endothelial dysfunction by reducing oxidative stress and activating NF-κB [67,97]. Currently, the effects of angiotensin-converting enzyme receptor 2 (ACE2), which is present in endothelial cells (EC) of the lung, heart, kidney, intestine, and in systemic vessels (small and large arteries, capillaries), are discussed and explored. In the current context, they become activated and dysfunctional in the case of severe acute respiratory syndrome caused by SARS-CoV-2 infection. As a result of endothelial activation and dysfunction, serum levels of proinflammatory cytokines (interleukin-1, interleukin-6 (IL -6), and tumor necrosis factor-α), von Willebrand factor (vWF) antigen, vWF activity, factor VIII, and chemokines (monocyte chemoattractant protein-1) are increased. Acute phase reactant levels are also elevated in SARS-CoV-2 infection (IL-6, D-dimers, C-reactive protein) were observed. Endothelial dysfunction is therefore hypothesized to contribute to pulmonary, renal, cardiovascular vascular inflammation, and coagulopathy associated with COVID-19 infection, particularly microemboli in alveolar capillaries [67,68,69].

Currently, there is some conflicting evidence about NF-κB activity between in vitro and in vivo studies. In vitro studies suggest that vitamin D reduces inflammation through NF-κB activity, although human clinical trials have not shown an effect of vitamin D supplementation on inflammatory markers or NF-κB activity in vivo. Recently, an updated systematic review with meta-analysis and meta-regression demonstrated that vitamin D treatment does not improve endothelial dysfunction [70,71,72,73]. Although data in the literature between in vitro and in vivo studies are conflicting, it is worth noting the methodological differences based on the results. In vivo, the effects of vitamin D supplementation are based exclusively on indices (central augmentation index, flow-mediated dilation, pulse wave velocity), which cannot be consistent with endothelial activation biomarkers used in clinical practice [75,97].

Further studies regarding the association between endothelial dysfunction in patients with COVID-19 and the benefits of vitamin D supplementation are needed to understand the therapeutic opportunities [32,75,76,79,105].

## 9. Conclusions

The specialized literature presents clear evidence regarding the role of serum vitamin D levels in patients with SARS-CoV-2 infection. Assessment of serum 25-OHD concentration is important for detailed identification of physiological effects of vitamin D with pathophysiological implications. Relationships between the immune system, respiratory infections, and the role of vitamin D in the context of COVID-19 infection have also been identified. At the same time, oral manifestations caused by vitamin D deficiency were noted. Many studies have been noted that have provided concrete evidence regarding the function of vitamin D in maintaining glucose tolerance. Epidemiological data and biochemical and immunological evidence have shown that vitamin D could be an important agent to modify the evolution of patients with COVID-19 infection.

## Figures and Tables

**Table 1:** The risk of vitamin D deficiency.

Study	Purpose	Comments
Fleet 2017, [26]	To establish that hormonal control of vitamin D will regulate serum calcium levels so that they are maintained within a very narrow range	The study showed the importance of vitamin D and signaling through the VDR in the control of intestinal calcium absorption, renal calcium metabolism, bone metabolism, and even vitamin D metabolism
Wacker 2013, [27]	To evaluate what blood level of 25-hydroxyvitamin D should be attained for both bone health and reducing risk for vitamin D deficiency associated acute and chronic diseases and how much vitamin D should be supplemented	The authors suggest a pivotal role of vitamin D for a plethora of physiological functions and health outcomes including neuropsychiatric disorders
Ghozali 2022, [28]	To report metabolic effects of vitamin D, in order to identify its potential use to prevent and overcome metabolic diseases	The study provided concrete evidence for the function of vitamin D in maintaining glucose tolerance
Braga 2017, [29]	To determine the effect of 1,25-D3 on myogenic cell differentiation in skeletal muscle derived stem cells	Vitamin D exerts a clear pro-myogenic effect on satellite cells in charge of muscle reconstitution after muscle injury or muscle waste
Pincombe 2019, [30]	To establish the effect of vitamin D supplementation on endothelial function	The authors have proven that vitamin D supplementation showed no improvement in endothelial function
Kheiri 2018, [31]	To correlate studies and trials on the effect of vitamin D supplementation on cardiovascular risk factors and hypertension	Low vitamin D is associated with hypertension and higher cardiovascular and all-cause mortality
Banerjee 2021, [32]	To elucidate how vitamin D through its diverse actions on immune effector cells could have a modulatory role on the pathogenic mechanisms of COVID-19	Epidemiological data and biochemical and immunological evidence showed that vitamin D could be an important disease modifying-agent in COVID-19

**Table 2:** Relationship between vitamin D and COVID-19.

Study	Purpose	Comments	Outcome
Annweiler 2020, [67]	to evaluate whether bolus vitamin D supplementation taken regularly was effective in improving survival among hospitalized frail elderly COVID-19 patients	in elderly vitamin D supplementation was associated with less severe COVID-19 and better survival.	Adjuvant treatment for COVID-19
Sulli 2021, [68]	to correlate the 25OH-vitamin D serum concentrations with clinical parameters of lung involvement, in elderly patients hospitalized for SARS-CoV-2 infection	25OH-vitamin D serum deficiency is associated with more severe lung involvement, longer disease duration and risk of death, in elderly COVID-19 patients.	Crucial risk factor at any age
Alexander 2020, [69]	to investigate the usefulness of early micronutrient intervention, focus on vitamin D, to relieve escalation of COVID-19	they recomend early outpatient nutritional intervention in SARS-CoV-2 exposed or high-risk subjects.	Nutritive adjuvant therapy
Radujkovic 2020, [70]	to explore possible associations of vitamin D status with disease severity and survival	Vitamin D deficiency was associated with higher risk of invasive mechanical ventilation and death, when adjusted for age, gender, and comorbidities.	High risk of potentially fatal COVID-19
Sarhan 2022, [71]	to explore the effect of high-dose intramuscular vitamin D in hospitalized adults infected with moderate-to-severe SARS-CoV-2 in comparison with the standard of care in the COVID-19 protocol.	study showed that high-dose vitamin D was considered a promising treatment in the suppression of cytokine storms among COVID-19 patients and was associated with better clinical improvement and fewer adverse outcomes compared to low-dose vitamin D.	Cytokine response against infections in the era of COVID-19
Pizzini 2020, [72]	to investigate associations of vitamin D status to disease presentation within the COVID-19 registry	Vitamin D deficiency is frequent among COVID-19 patients but not associated with disease outcomes.	Disturbed parathyroid-vitamin-D axis
Hastie 2021, [73]	to establish whether baseline serum 25(OH)D concentration was associated with COVID-19 mortality, and inpatient confirmed COVID-19 infection, in UK Biobank participants	not a potential link between 25(OH)D concentrations and risk of severe COVID-19 infection and mortality.	25(OH)D and confirmed COVID-19 infection or mortality
Sabico 2021, [74]	to determine the effects of 5000 IU versus 1000 IU daily oral vitamin D supplementation in the recovery of symptoms and other clinical parameters among mild to moderate COVID-19 patients with sub-optimal vitamin D status	a 5000 IU daily oral vitamin D3 supplementation for 2 weeks reduces the time to recovery for cough and gustatory sensory loss among patients with sub-optimal vitamin D status and mild to moderate COVID-19 symptoms.	Vitamin D supplementation among those with suboptimal levels against COVID-19
Gönen 2021, [75]	to establish an acute treatment protocol to increase serum vitamin D, evaluate the effectiveness of vitamin D3 supplementation, and reveal the potential mechanisms in COVID-19	Vitamin D treatment shortened hospital stay and decreased mortality in COVID-19 cases.	Vitamin D supplementation is effective on various targeted parameters
Rustecka 2021, [76]	to evaluate whether home confinement led to decreased vitamin D serum levels in children in Warsaw, Poland.	The COVID-19 pandemic restrictions led to a significant decrease in vitamin D serum levels in children.	The importance of vitamin D supplementation in the paediatric population
Boulkrane 2020, [61]	To establish the potential role of vitamin D in SARS-CoV-2 virus/COVID-19 disease	The higher concentrations of vitamin D3 is better for the protection from various viral and respiratory infections.	Supplementation of vitamin D3 in COVID-19
Bishop 2020, [77]	To provide an update on current understanding of the prominent immune actions of vitamin D, as well as highlighting new, less well-recognized immune effects of vitamin D	There are strong evidence that vitamin D metabolic enzymes are expressed in virtually all cells in the innate and adaptive arms of the immune system.	Regulation of the NF-κB pathway during infection
Albergamo 2022, [78]	To explore the correlation studies between vitamin D deficiency and increased risks of severe COVID-19 disease and, similarly, between vitamin D deficiency and acute respiratory distress syndrome	Numerous studies highlight its immunomodulatory and anti-inflammatory properties, and its use was proposed in COVID-19.	Decreasing the severe symptoms due to inflammation and oxidative stress
Ghelani 2021, [79]	To consolidate the research surrounding the role of vitamin D in the treatment and prevention of COVID-19.	This current study shows evidence wich supports the links between vitamin D and COVID-19 and the benefits of vitamin D supplementation.	The toxicity of Vitamin D supplementation is far outweighed by the potential benefits in relation to protection against COVID-19.
Biesalski 2020, [80]	To establish whether an inadequate vitamin D supply has an influence on the progression and severity of COVID-19 disease	Various non-communicable diseases (hypertension, diabetes, CVD, metabolic syndrome), are associated with low vitamin D plasma levels.	Vitamin D deficiency increase the risk of severe COVID-19
Verdoia M 2021, [81]	To determine the potential implications for COVID-19 pandemic	Vitamin D appears to reduce the acute-phase response associated to a larger pulmonary damage and complications as ARDS or sepsis.	Vitamin D modulates the activity of IL-6
Weir 2020, [82]	To establish if vitamin D supplement would offer a relatively easy option to decrease the impact of the pandemic	Vitamin D appears to have beneficial effects against COVID-19, it would follow that the severity of the disease should lessen in the Northern hemisphere.	Vitamin D, an easy option to decrease the impact of the pandemic of COVID-19
Hribar 2020, [83]	To determine the relationship between vitamin D, PD, and COVID-19.	Vitamin D may have antiviral properties and play a role in protecting against infections, including respiratory illnesses.	2000–5000 IU/day of vitamin D3 in individuals with PD may be beneficial in reducing the risk and severity of COVID-19.

## Data Availability

Not applicable.
